# Mentorship and growth: Reflections from the Asian Medical Students' Association Competition in Japan

**DOI:** 10.51866/mol.733

**Published:** 2024-10-21

**Authors:** Abu Hassan Hasliza, Jin Xiang Leong, Ri Hong Ting, Wei Quan Heng

**Affiliations:** 1 MBBS, MMed, Department of Primary Care Medicine, National Defence University of Malaysia, Kem Sungai Besi, Kuala Lumpur, Malaysia. Email: hasliza@upnm.edu.my; 2 Medical Student, Faculty of Medicine and Defence Health, National Defence University of Malaysia, Kuala Lumpur, Malaysia.; 3 Medical Student, Faculty of Medicine and Defence Health, National Defence University of Malaysia, Kuala Lumpur, Malaysia.; 4 Medical Student, Faculty of Medicine and Defence Health, National Defence University of Malaysia, Kuala Lumpur, Malaysia.

**Keywords:** Disaster medicine, Education, Medical, Mentoring

Participating as a supervisor in the Asian Medical Students’ Association (AMSA) Competition in Japan was both enlightening and enriching. This prestigious event, which aimed to enhance medical students’ academic knowledge, cultural understanding and interpersonal skills, provided me with the opportunity to mentor aspiring professionals and reflect on my development as an educator. The AMSA Conference and Competition, held from 21 to 27 July 2024 at the International University of Health and Welfare’s Narita and Tokyo campuses, focused on the theme of ‘Disaster Medicine’. AMSC 2024 offered medical students a platform to explore disaster medicine, fostered international collaboration and idea exchange and highlighted the need for disaster preparedness and global health cooperation among future healthcare leaders.

From the beginning, the event was filled with great enthusiasm from participants all over Asia. It gave me great pleasure to witness such dynamic and progressive interactions between medical students from diverse cultural and linguistic backgrounds. The whole event transcended the norms of conventional medical education that we were all accustomed to.

Watching my Malaysian students present their research entitled “Ensuring Children’s Health Rights Amidst Armed Conflicts: A Review of Malnutrition and Utilization of Innovative Technological Approaches in Humanitarian Assistance” was a moment of immense pride. My Year 5, Year 2 and Year 1 students eloquently addressed the complex topic, demonstrating their deep understanding and resilience through clear delivery and their capability of handling tough questions during the Q&A session, all while showcasing a strong sense of camaraderie among themselves.

My roles as a supervisor varied, including providing guidance, offering constructive feedback and practising active listening. This journey began before *Hari Raya Puasa* in April 2024, during which we managed various responsibilities, including teaching and personal duties. We often held discussions on weekends, either in person or online, to accommodate everyone’s schedules. Balancing support while allowing students the freedom to explore their ideas was a challenging yet rewarding aspect of mentorship.

The competition featured remarkable projects from international participants, highlighting their skills and dedication. Seeing my students transform from tentative participants to confident presenters was immensely satisfying and filled me with pride. This experience reinforced the profound impact of mentorship, strengthened my passion for teaching and emphasised the importance of supporting the next generation of medical professionals.

Although we did not win any prizes, the experience was priceless. The knowledge-sharing sessions with students from the Asian region provided rich learning opportunities beyond the competition. The AMSA Competition in Japan was more than just an event; it was a blend of ideas, cultures and experiences that left a lasting impact. As I return to my university, I carry with me the lessons learnt and the memories created. I look forward to future opportunities to mentor students, appreciating the insights gained both locally and internationally. Additionally, exploring Japan’s beauty, modernisation, cleanliness and cultural heritage has enriched our reflections on our own values and cultures.

I would like to express my deepest gratitude to my cherished students, their parents and the Faculty of Medicine and Defence Health at the National Defence University of Malaysia for their unwavering support of our project and this event.

**Figure 1:**
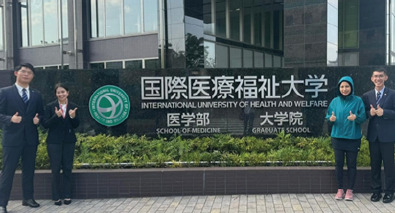
Narita campus

**Figure 2:**
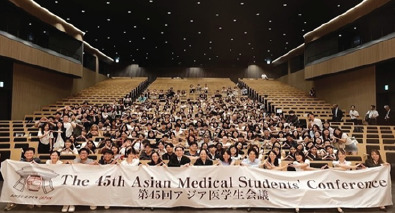
Tokyo campus

**Figure 3:**
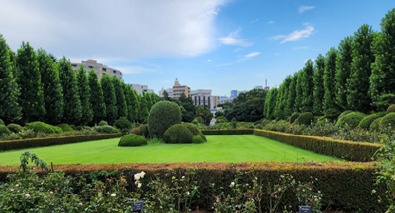
Shinjuku Gyoen National Garden

**Figure 4:**
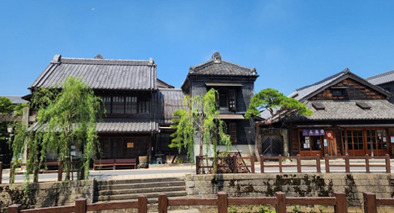
Cultural heritage in Sawara town

**Figure 5:**
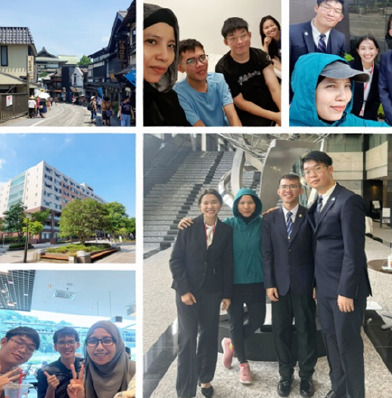
Unforgettable journey

